# Highly efficient hierarchically porous carbon-silica composite for sub-terahertz stealth and shielding applications

**DOI:** 10.1016/j.csbj.2025.02.021

**Published:** 2025-02-25

**Authors:** Nikolaos Xenidis, Aleksandra Przewłoka, Konrad Godziszewski, Łukasz Osuchowski, Krystian Pavłov, Aleksandra Krajewska, Yevhen Yashchyshyn, Zygmunt Mierczyk, Joachim Oberhammer, Dmitri Lioubtchenko

**Affiliations:** a Division of Micro and Nanosystems, KTH Royal Institute of Technology, Malvinas Väg 10, Stockholm, SE-100 44, Sweden; b CENTERA, Institute of High Pressure Physics, PAS, 29/37 Sokołowska Street, Warsaw, 01-142, Poland; c Institute of Optoelectronics, Military University of Technology, gen. Sylwestra Kaliskiego 2, Warsaw, 00-098, Poland; d Institute of Radioelectronics and Multimedia Technology, Warsaw University of Technology, Nowowiejska 15/19, Warsaw, 00-665, Poland; e Centre for Advanced Materials and Technologies (CEZAMAT), Warsaw University of Technology, Poleczki 19, Warsaw, 02-822, Poland

**Keywords:** Shielding, Porous carbon, Terahertz, Stealth

## Abstract

The development of future 6G communication systems necessitates advanced materials for efficient electromagnetic interference shielding in the sub-terahertz frequency range. This study presents the preparation, porosimetry analysis, compositional and electromagnetic characterization of a highly efficient hierarchically porous carbon-silica composite suitable for shielding and stealth applications in this frequency regime. The composite, fabricated using a mixture of carbon powder and tetraethoxysilane, possesses a highly porous structure with high surface area, which facilitates multiple reflections and scattering of electromagnetic waves. Electromagnetic characterization was conducted using a free-space semi-optical method at 140-220 GHz, focusing on reflection-only measurements due to the sample's large thickness. The results demonstrate that the composite exhibits a qualified bandwidth of 83% over the measured frequency band, with a maximum reflection loss of 35 dB at 187 GHz. Furthermore, measurements demonstrate that electromagnetic power within the sample's volume is effectively attenuated. The composite's shielding efficiency due to reflection is on average 0.26 dB across the band, highlighting its potential for high frequency EMI shielding and stealth applications.

## Highlights

• Novel porous carbon-silica composite for sub-terahertz EMI shielding and stealth applications.
• 83% Qualified bandwidth in the 140-220 GHz frequency band.
• Average shielding effectiveness due to reflection averages 0.26 dB across the band.

## Graphical abstract

**Figure:**
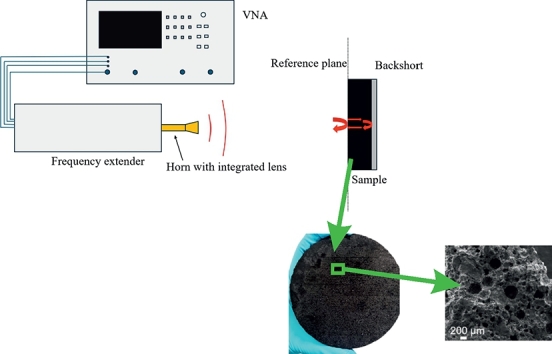

## 1 Introduction

The fifth generation (5G) of mobile wireless communication has only recently become reality, offering communication links with higher data rates and lower latency. However, to sustain the ever increasing number of interconnected devices, the vision of 6G has emerged, expected to be developed within the next decade [1]. Beyond-5G networks are expected to achieve data rates of up to 1 Tbit/s and enable connectivity for billions of devices. To handle this amount of data, 6G will use upper millimeter-wave and sub-terahertz (sub-THz) frequencies.

As the frequency of communication systems increases, the challenge of managing electromagnetic interference (EMI) becomes more critical. EMI can manifest as unwanted noise, disrupting electronic systems and degrading their performance. More alarmingly, EMI can serve as a conduit for unauthorized data transmission, posing significant security risks. Thus, the development of effective electromagnetic (EM) wave shielding and absorbing materials is of paramount importance [2], [3].

Ideally, an absorbing material acts as a black body, both blocking the transmission of incident waves for shielding and minimizing reflections for stealth performance. These requirements, however, are often contradictory because materials with high attenuation often exhibit high reflectivity due to high impedance mismatches. Traditionally, EMI shielding has employed techniques such as isolation with metallic foils or conductive coatings [4]. However, modern applications demand materials that provide broadband absorption while minimizing reflection. Such materials leverage multiple mechanisms of loss, including electrical and magnetic dissipation, to achieve wideband effectiveness. Additionally, it is desirable that the absorbers are lightweight, thin, functional in a wide range of temperatures and cost effective.

Nanomaterials have emerged as promising candidates for EMI shielding due to their unique electrical, magnetic and structural properties [5], [6], [7]. Graphene [8], [9], [10], single- and multi-walled carbon nanotubes (CNTs) [11], [12], Mxenes [13] have demonstrated effective EMI shielding capabilities, especially at higher frequencies.

Among these materials, porous carbon-based structures have shown considerable promise in EM absorption and shielding applications [14], [15], [16], [17]. Their porous architecture increases the surface area, facilitating multiple reflections and scattering of EM waves and therefore promoting energy dissipation, while keeping the material lightweight. They also offer a sustainable solution, since they can be derived from biomass, and generally they display excellent environmental stability e.g. in high temperatures, which are often required in semiconductor processing.

However, the absorption performance of such materials so far has mostly been investigated in microwave frequencies below 20 GHz, whereas studies in sub-THz frequencies are sparse. Accurate electromagnetic characterization in this frequency range is challenging, as it requires specialized equipment and techniques. At these frequencies, skin-depths, scattering from micro/nanostructures and dielectric/conductive losses can differ considerably, making simultaneous shielding and stealth performance more challenging. Consequently, developing and testing novel absorbers for sub-THz frequencies is crucial to meet the high-bandwidth needs of future communication systems. In this study, we investigate the stealth and shielding capabilities of a novel carbon-silica composite at sub-THz frequencies. This material, characterized by its high surface area due to its porous structure and the added mechanical and thermal stability from the inexpensive silica binder meets the requirements for high frequency broadband EMI shielding across the 140-220 GHz band. The remaining of this paper discusses the fabrication of the sample and its characterization using scanning electron microscopy (SEM), Raman spectroscopy, X-ray photoelectron spectroscopy (XPS), energy-dispersive X-ray spectroscopy (EDS), porosimetry analysis and quasi-optical electromagnetic characterization. X-ray diffraction (XRD) data is also included in supplementary material.

## 2 Materials and methods

### 2.1 Sample preparation

**Fig. 1:**
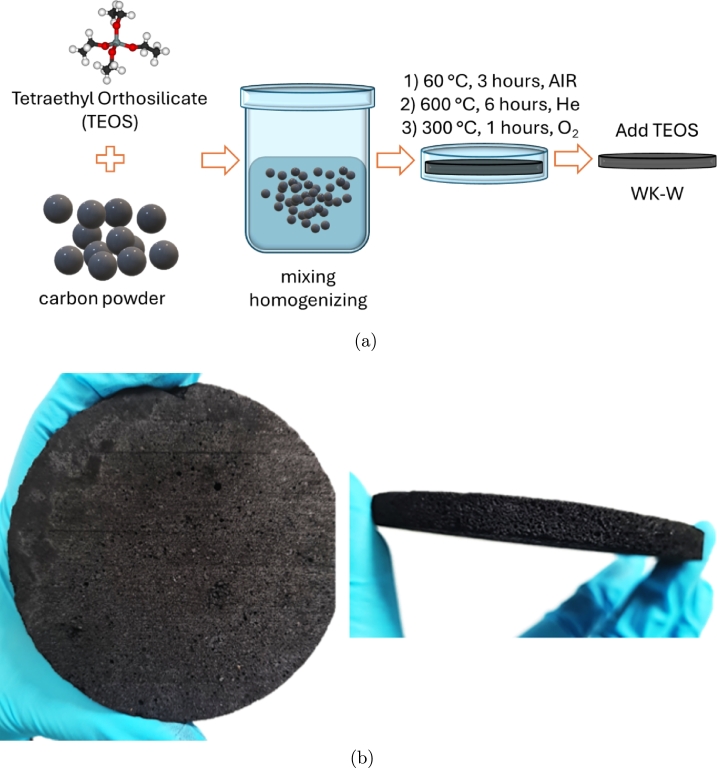
Fabrication process of the carbon-silica sample **(a)** and image of the final disk-shaped sample after fabrication **(b)**.

The fabricated carbon-silica composite is a mixture of carbon powder and organo-silica reagent that acts as adhesive substance (binder). 200 grams of waste-derived carbon powder (GEMINUS sp. z o.o., Wola Ducka Poland) and 100 ml of tetraethoxysilane (TEOS) (Chempur, Poland) were mixed. In the first step, the carbon powder was purified using an ethanol-acetone-water mixture in the Soxhlet apparatus. Afterwards, carbon was mixed with TEOS and transferred to a form. The resulting mixture was slowly heated to 60 °C and maintained at this temperature for 3 hours. Subsequently, the composite was carbonized in oxygen-free atmosphere using flowing helium at 600 °C for 6 hours in a tube furnace. After carbonization, the mixture was purified in an oxygen atmosphere at 300 °C for 1 hour. The whole process of chemical synthesis and formation was performed in a flat mold, resulting in a plate-shaped sample. Once cooled, TEOS was added in portions to completely cover carbon particles, thereby enhancing adhesion throughout the mass and minimizing cracks. Next, 40 ml of ethyl alcohol was added to the mixture, stirred vigorously and sealed to ensure cross-linking of silica. Finally, the carbon-silica composite was removed from the mold and dried for 48 hours in air. The final sample has a thickness of 7.52 mm and a diameter of 89 mm. Fig. 1a shows the fabrication process in steps and Fig. 1b shows the final porous, disk-shaped sample.

### 2.2 Scanning electron microscopy

The visualization of the surface morphology of the carbon-silica composite was carried out using Carl Zeiss Auriga 60 scanning electron microscope in a high vacuum mode using Everhart–Thornley secondary electron detector. As a trade-off between sample charging, resolution, and working distance, an accelerating voltage range of 1.5 kV to 5 kV was chosen.

### 2.3 Porosimetry analysis

#### 2.3.1 Nitrogen adsorption–desorption

The porosity of the carbon-silica composite was analyzed by low temperature nitrogen adsorption at -196 °C (77 K). Before adsorption measurements, the hybrid structure was degassed in standard conditions, i.e. in high vacuum and 200 °C. Low-temperature (-196 °C) adsorption-desorption nitrogen isotherms were measured using the automatic analyzer ASAP 2020 (Micromeritics Instrument Corp., Norcross, GA, USA). The Brunauer-Emmett-Teller (BET) method [18], [19] in standard relative pressure range $p/p_{0}$ from 0.05 to 0.2 was used to evaluate the specific surface area. Furthermore, the total pore volume ($V_{t}$) was calculated by converting the adsorbed nitrogen volume at a relative pressure of ∼0.99 $p/p_{0}$ to the volume of liquid nitrogen at the temperature of the experiment. The pore size distribution (PSD) was determined based on the nitrogen adsorption data using density functional theory (PSD-DFT) implemented in the analyzer program ASAP 2020 V3.01.

#### 2.3.2 Mercury intrusion porosimetry

Mercury intrusion porosimetry (MIP) was performed with the Micromeritics AutoPore IV 9500 apparatus. Initially, the sample was degassed at 110 °C for 1 hour and then at 25 °C for 30 minutes to a pressure of 50 μm Hg. Mercury intrusion was performed by incrementally increasing the applied pressure from 0.05 MPa to 414 MPa, forcing mercury into the pores. The effective pore radius *r* was calculated based on the Washburn equation,

(1) $$r=-\frac{2\sigma \cos(\theta )}{\Delta p}$$

where *σ* is the surface tension of mercury (assumed 0.480 N/m for carbon-based materials), *θ* is the contact angle of the solid surface for mercury (assumed 2.478 rad for carbon-based adsorbents) and Δ*p* is the applied pressure.

### 2.4 Raman spectroscopy

Raman spectroscopy was carried out at room temperature and in the ambient environment using a Renishaw InVia micro-Raman (Renishaw, Wotton-under-Edge, UK) system equipped with the Nd: YAG laser with an excitation wavelength of 532 nm and power of 2.4 mW on the sample. The laser beam power was focused on the samples via a 100x magnification objective lens, using an exposure time of 10 s and a single accumulation per measurement.

### 2.5 X-ray photoelectron spectroscopy

X-ray photoelectron spectroscopy was performed with a Physical Electronics Quantera II Scanning XPS Microprobe with an Al Kα monochromatic X-ray source and a 100 μm beam diameter. The survey spectra between 0 and 1400 eV were recorded with 0.8 eV resolution.

### 2.6 Energy dispersive X-ray spectroscopy

Energy dispersive X-ray spectroscopy was performed with a Hitachi S-3400N scanning electron microscope paired with Thermo UltraDry detector. A small piece of the sample was cut and the inner surface was analyzed. Measurements were performed with 10 kV accelerating voltage and 10 mm working distance. In order to eliminate artifacts, additional reference measurement was performed on the aluminum sample stage.

### 2.7 Electromagnetic characterization

**Fig. 2:**
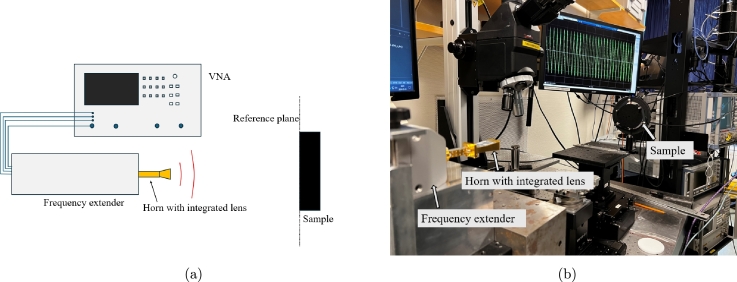
Schematic of the measurement setup, showing the VNA and the frequency extender feeding the antenna illuminating the sample **(a)**, and the actual measurement setup **(b)**.

**Fig. 3:**
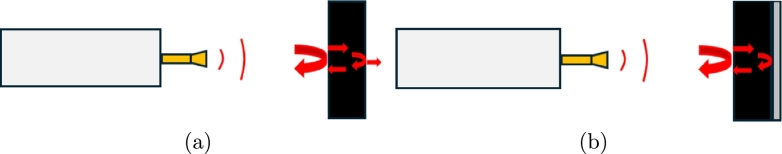
Schematic of measurement A, where the sample (black) is backed by free space **(a)**, and measurement B, where the sample is backed by metal (grey) **(b)**.

Electromagnetic characterization in the sub-THz range was performed using a free space quasi–optical measurement setup, as shown in Fig. 2. More specifically, Fig. 2a shows the schematic of the measurement, in which a vector network analyzer (VNA, Rohde & Schwarz ZVA-24) was connected to a frequency extender unit at 140-220 GHz (Rohde & Schwarz ZC220). One port calibration using short, offset short and match standards was performed at the flange of the extender to correct for systematic errors. A horn antenna with integrated plano-convex lens (Elmika Ltd, Vilnius, Lithuania) of 27 dBi gain illuminating the sample was connected to the frequency extender and then a second calibration (short, offset short, match) was performed to bring the reference plane at the interface of the sample under test. For the second calibration, a gold-coated wafer providing maximum reflectivity was used as calibration standard. The wafer was placed on a moving stage with micrometer accuracy to account for the offset short standard and was tilted 45^∘^ with respect to the antenna axis for the match standard. Commercial laser (Thorlabs, Inc) mounted on the waveguide flange was used for precise alignment of all components. Time gating was applied afterwards to correct for residual errors. Fig. 2b shows the actual setup in the lab. Two measurements were performed. In the first measurement, the sample is backed by free space and its reflectivity is measured (measurement A, Fig. 3a). In the second measurement, the sample is backed by a metalized wafer placed right behind the sample (measurement B, Fig. 3b).

## 3 Results and discussion

### 3.1 Scanning electron microscopy

**Fig. 4:**
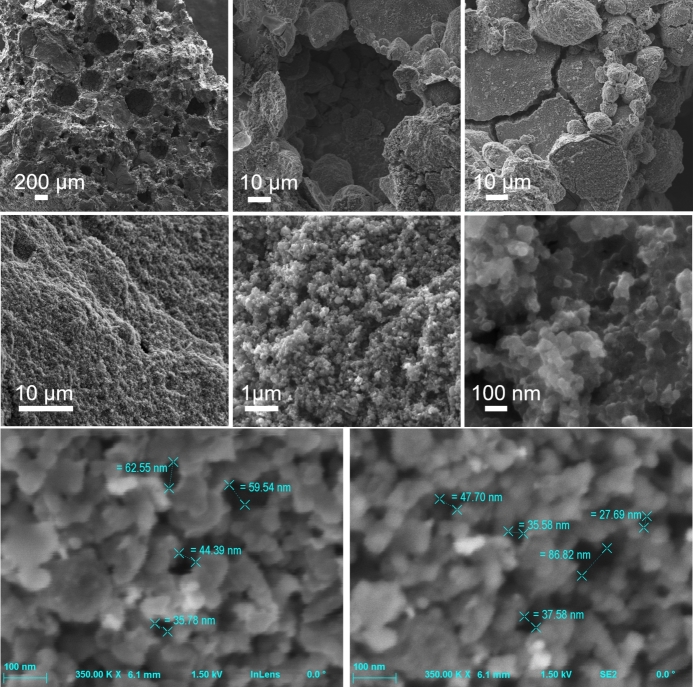
SEM images of the composite at different magnifications. Higher magnifications reveal nanoscale pores.

As the SEM images of the composite reveal in Fig. 4, the sample is characterized by well-developed porosity and larger magnifications show a grain–like morphology. This structure is repeatable throughout the whole volume of the sample, even at a nanometer level. This morphological evidence is consistent with the high specific surface area measured by porosimetry analysis (Section 3.2). High magnification images in Fig. 4 reveal approximate sizes of nanoscale pores in the range of 28 to 86 nanometers.

### 3.2 Porosimetry analysis

**Fig. 5:**
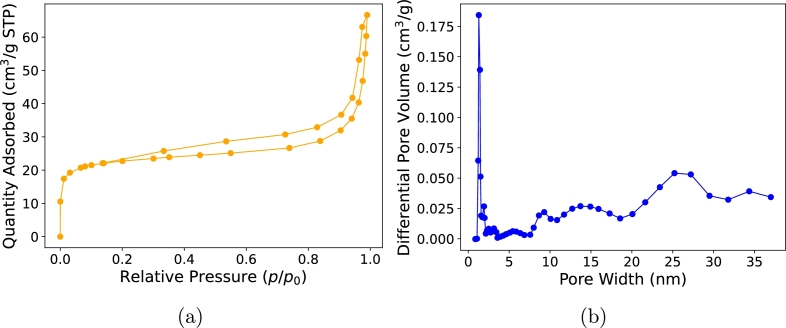
Low temperature (77 K, -196 °C) nitrogen adsorption-desorption isotherm **(a)**, and differential pore volume distribution functions **(b)** of the composite.

Low-temperature nitrogen adsorption-desorption isotherm for the sample is presented in Fig. 5a. According to the IUPAC classification [20], the shape of the obtained nitrogen adsorption-desorption isotherm is categorized as a IV type with a hysteresis loop B [20], [21], [22]. Functions of this type are characteristic of mesoporous materials. Using the BET method for data in the relative pressure range 0.05-0.2 [19], the composite is found to have a specific surface area of 66.6 m^2^/g, and the total pore volume, estimated at a relative pressure of ∼0.99 $p/p_{0}$, is $V_{t}=0.09$ cm^3^/g. Analysis of the pore size distribution via density functional theory [19] reveals a multi-peak distribution within the 0–40 nm range, as presented in Fig. 5b. While nitrogen adsorption-desorption accurately captures pores up to around 40 nm, additional methods are needed for capturing larger pores. Complementary measurements were performed using mercury intrusion porosimetry to further investigate the pore structure, particularly in the meso- to macropore range. The structural parameters obtained are summarized in Table 1.

**Table 1:** MIP porosimetry data for the carbon-silica composite.

Apparent Density (g/cm^3^)	True Density (g/cm^3^)	Total Intrusion Volume (cm^3^/g)	Total Pore Area (m^2^/g)	Total Porosity (%)
0.836	1.263	0.405	43.7	33.9

**Fig. 6:**
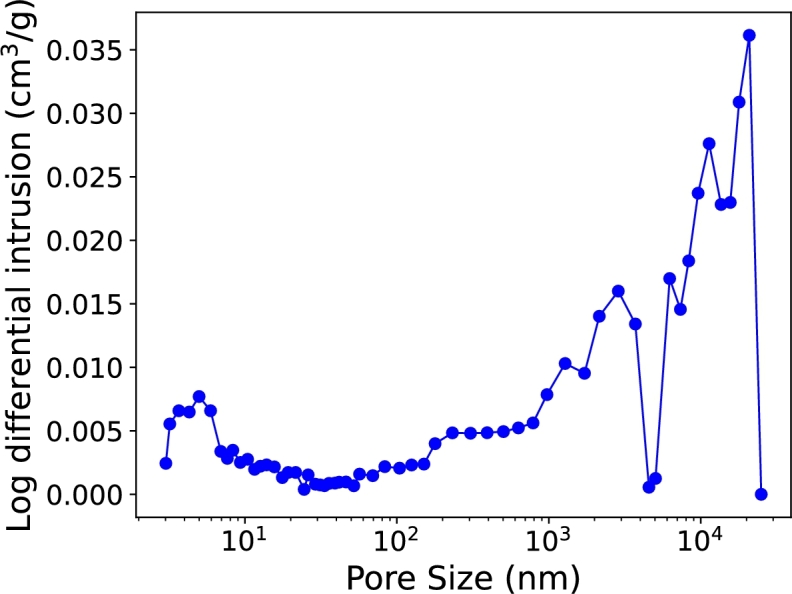
Pore size distribution measured by mercury intrusion.

MIP results of Fig. 6 reveal a subtle but measurable nanoporosity under ∼ 20 nm, which correlates well with nitrogen adsorption-desorption observations. MIP also reveals an intensified porosity of larger pore volumes spanning from 100 nm to several micrometers. This is consistent with SEM imaging observation of larger voids in Sec. 3.1. This broad pore size distribution or “quasi-fractal” morphology creates multiple scattering paths and prolongs wave propagation within the material, therefore enhancing its absorbing/lossy properties. The synergy of fine pores (as measured by nitrogen adsorption-desorption) and larger pores (as measured by MIP) is expected to contribute to a very broadband operation of the absorber, especially at higher frequencies where the wavelength becomes comparable with the pore sizes [23], [24], [25], [26], [27], [28], [29].

### 3.3 Raman spectroscopy

**Fig. 7:**
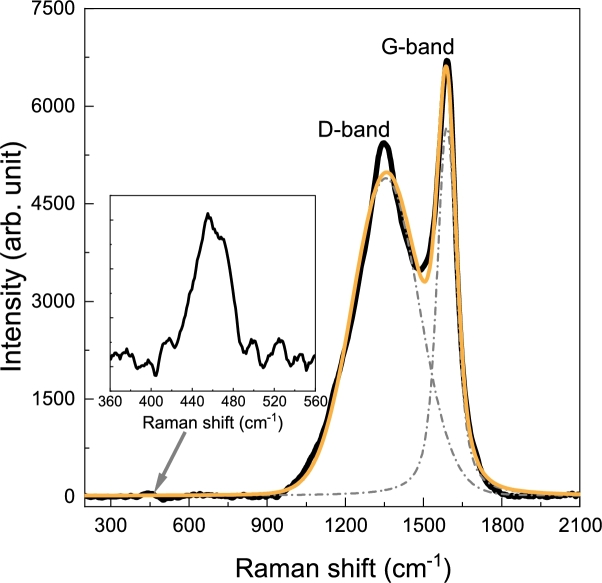
Representative Raman spectra of the sample. The black curve shows the spectra line before fitting. The gray dashed curves represent the individual spectral components obtained via Lorentzian deconvolution. The yellow curve is the overall fitted spectrum. Inset: the zoom-in view of the low-frequency region.

Raman spectrum of the analyzed composite presented in Fig. 7 clearly reveals two dominant peaks assigned to the typical amorphous carbon [30], [31], [32], [33], [34], [35]. In order to improve the accuracy in the determination of spectroscopic parameters, a curve deconvolution was performed using the Lorentzian function. The D-band, observed at ∼1350 cm^-1^, corresponds to the appearance of the structural defects in the hexagonal sp^2^ carbon lattice as well as to the edge defects, while the G-band located at ∼1589 cm^-1^ is attributed to the in-plane vibration of the sp^2^ carbon atoms [34], [35], [33]. The measured I_D_/I_G_ ratio of the composite is approximately 0.75. Moreover, the spectrum of the sample also contains another broad peak located between 400 and 500 cm^-1^, whose appearance may be associated with a contribution from amorphous Si–O–C-related structures [36], [37]. Similar observations at the low-frequency region for composites based on TEOS and carbon were made by T. Minamisawa et al. [38] and S.Y. Kim et al. [39].

### 3.4 X-ray photoelectron spectroscopy

**Fig. 8:**
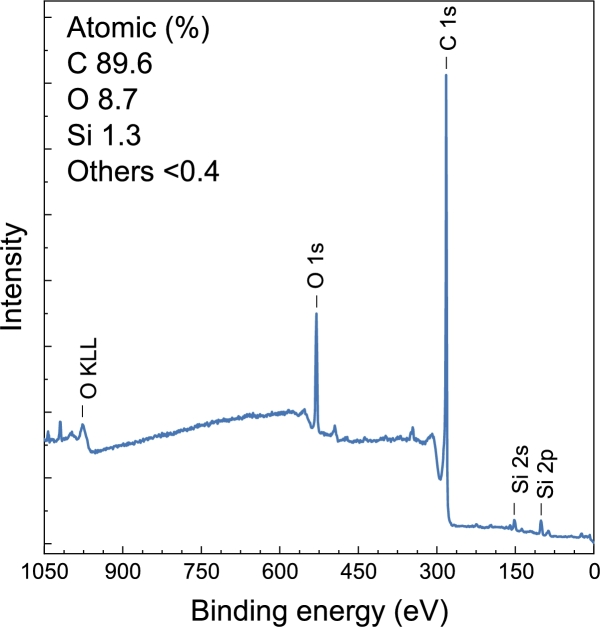
XPS survey spectrum of the sample.

XPS analysis was performed to examine the surface elemental composition of the composite, and confirms the successful integration of silica within the carbon matrix. The survey XPS spectrum presented in Fig. 8 reveals prominent photoelectron peaks for Si2p, C1s, and O1s, indicating the presence of silicon, carbon, and oxygen in the sample. This consistency with the expected composition indicates the chemical stability of the composite structure. These results can be correlated with the Raman spectroscopy findings, which indicate the presence of structural disorder in the carbon matrix (as shown by the D and G bands).

### 3.5 Energy dispersive X-ray spectroscopy

**Fig. 9:**
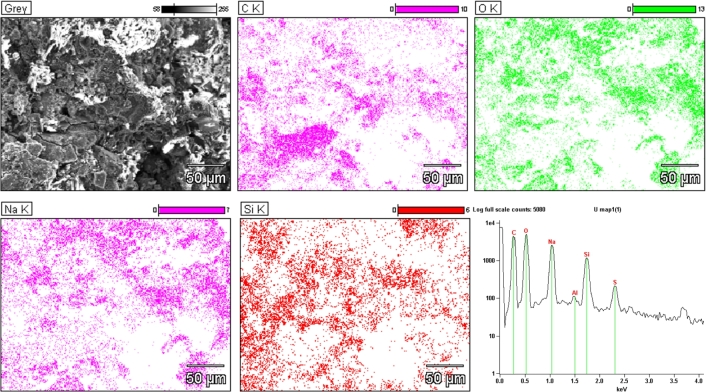
SEM image, element distribution maps and EDS spectrum of the sample.

EDS analysis was performed to examine the elemental composition of the sample and assess the uniform dispersion of its components. An averaged elemental map from an arbitrary area of the sample was also obtained by gathering detection counts from the whole scanning electron microscope's field of view at 400x magnification and 15 kV accelerating voltage. This data was then quantified in order to extract the material's elemental ratios (Table 2). Fig. 9 shows that the signals corresponding to oxygen (O), silicon (Si), and carbon (C) are uniformly distributed throughout the sample. In addition to these expected elements, extra peaks were observed during the measurements that were absent in a reference measurement, thereby ruling out chamber contamination as a potential source. Aluminum (Al) and magnesium (Mg) signals are present because of the specimen stage. Sodium (Na) and sulfur (S) signals might be explained by impurities since the source carbon powder is waste-derived. High intensity of the 1.05 keV peak might also be strongly contributed by double absorption of 0.525 keV oxygen-related K*α* photons, which creates a sum peak. This hypothesis is supported by the fact that O and Na distribution maps are very similar.

**Table 2:** Elemental composition determined by EDS averaged over the SEM field of view.

Element line	C K	O K	Na K	Si K	Mg K	S K
Weight %	34.9	42.5	13.6	7.2	0.1	1.6
Atom %	44.9	41.1	9.1	4.0	0.1	0.8

### 3.6 Electromagnetic characterization

Due to the structure of the sample, high frequency waveguide measurements are challenging, prohibiting direct integration inside the waveguide cavity. Therefore, a free space semi-optical method is chosen, as explained in section 2.7. The large thickness of the sample does not allow for full characterization of two-port measurements utilizing both reflection and transmission, due to the limited dynamic range of the instruments. Therefore, only reflection measurements are used. According to the method described in [40], it is possible to fully characterize the sample by using reflection-only measurements. However, this method relies heavily on very accurate placement of the sample to the reference plane, and any small deviation from this position will create accumulative phase inaccuracies that will alter the extracted complex positivity significantly. It is noted that small deviations from the reference plane affect primarily the phase and do not have significant impact on the magnitude of the reflection parameter $S_{11}$. Therefore, two measurements are provided: in the first one (measurement A, see Fig. 3a), the sample is free from both sides, resembling a lossy Fabry-Perot cavity illuminated by a plane wave. In the second measurement (measurement B, see Fig. 3b), the sample is backed by a reflective metallic plate. For measurement A, a primary reflection occurs at the first interface of the sample. Then, part of the power is absorbed by the sample, and part of it is transmitted through the sample away from the source as it exits the second interface of the sample. For measurement B, no power can be transmitted away from the source because of the metallic backshort, which reflects back all power reaching the second interface. By comparing the reflection from these two measurements, we can qualitatively assess the absorption of - or equivalently the transmission through - the sample. In fact, these two measurements are enough to even accurately calculate transmission through the sample [41].

**Fig. 10:**
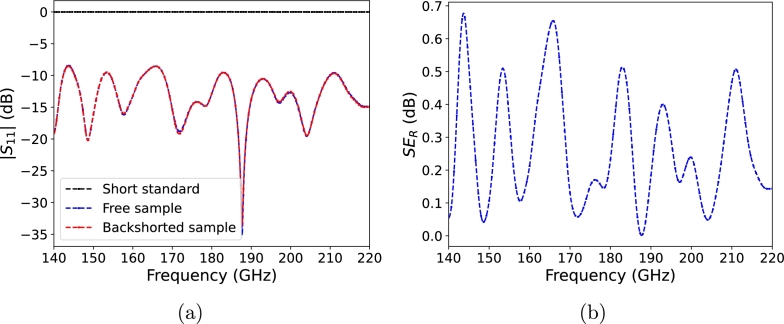
Reflection data of the sample (free and backshorted). The short calibration standard is also given for reference **(a)** and shielding efficiency due to reflection **(b)**.

Fig. 10a shows the reflection parameter $\left|S_{11}\right|$ (dB), measured at the sample's interface, for both the free and the backshorted sample. For reference, the measured short calibration standard (gold plated wafer used for calibration) is also given. These results indicate that the sample has a qualified bandwidth (for which $|S_{11}|<-10$ dB) of 83% over the measured frequency band, with a maximum reflection loss of 35 dB at 187 GHz. Furthermore, there is no detectable difference in reflection between the two measurements of the free sample and the backshorted sample. That means reflection occurring at the second backshorted interface in measurement B is extremely weak compared to the primary reflection occurring at the first interface, and cannot be detected. Therefore, while we cannot quantify the actual transmission (or equivalently, absorption) through the sample, we can safely conclude that all power traveling within the sample has been completely attenuated. Consequently, the sample provides absorption-dominant shielding. Shielding efficiency due to reflection, defined as $\text{SE}{}_{\text{R}}=-10\log_{10}(1-|S_{11}{|}^{2})$ [42] is given in Fig. 10b, with an average ${\text{SE}}_{\text{R}}$ of 0.26 dB across the whole band.

## 4 Conclusions

This study presented the preparation, compositional, porosimetric and electromagnetic characterization of a novel porous carbon-silica composite, suitable for sub-THz shielding applications. Porosimetric analysis reveals that the composite possesses a significant hierarchical porous structure, which is beneficial for multiple reflections and scattering of EM waves, thereby promoting losses. EM characterization was conducted using a free space semi-optical method due to the sample's structure, which prohibits direct integration inside a waveguide cavity. Reflection-only measurements were employed to fully characterize the sample. The results indicate that the investigated composite is an excellent candidate for broadband stealth and shielding applications in future high frequency communication systems. Subsequent research will focus on thickness dependency studies and size reduction to enhance compatibility with compact systems, higher frequency investigation, and incorporation of transmission measurements for a complete shielding analysis.

## CRediT authorship contribution statement

**Nikolaos Xenidis:** Writing – review & editing, Writing – original draft, Visualization, Validation, Software, Methodology, Investigation, Formal analysis, Data curation, Conceptualization. **Aleksandra Przewłoka:** Writing – original draft, Investigation, Data curation. **Konrad Godziszewski:** Validation. **Łukasz Osuchowski:** Writing – review & editing, Writing – original draft, Methodology, Investigation, Formal analysis. **Krystian Pavłov:** Data curation, Investigation, Validation, Writing – original draft, Writing – review & editing. **Aleksandra Krajewska:** Investigation, Data curation. **Yevhen Yashchyshyn:** Validation. **Zygmunt Mierczyk:** Resources, Funding acquisition. **Joachim Oberhammer:** Funding acquisition, Resources. **Dmitri Lioubtchenko:** Supervision.

## Declaration of Competing Interest

The authors declare that they have no known competing financial interests or personal relationships that could have appeared to influence the work reported in this paper.

## Appendix A Supplementary material

MMC Supplementary material includes additional SEM images and XRD analysis of the sample. MMC
