# Supplementary material for: Highly efficient hierarchically porous carbon-silica composite for sub-terahertz stealth and shielding applications
Source: Comput Struct Biotechnol J. 2025 Feb 25;29:52–9. doi: 10.1016/j.csbj.2025.02.021 (PMC11930209; doi:10.1016/j.csbj.2025.02.021)
Supplement: MMC — Supplementary material includes additional SEM images and XRD analysis of the sample. [file mmc1.pdf]

Supporting Information

Highly Efficient Hierarchically Porous Carbon-Silica Composite for sub-Terahertz Stealth and Shielding Applications

Nikolaos Xenidis, Aleksandra Przewłoka, Konrad Godziszewski, Łukasz Osuchowski, Krystian Pavłov, Aleksandra Krajewska, Yevhen Yashchyshyn, Zygmunt Mierczyk, Joachim Oberhammer, Dmitri Lioubtchenko

1. Additional SEM images

Porosity of different scales can be observed in these images:

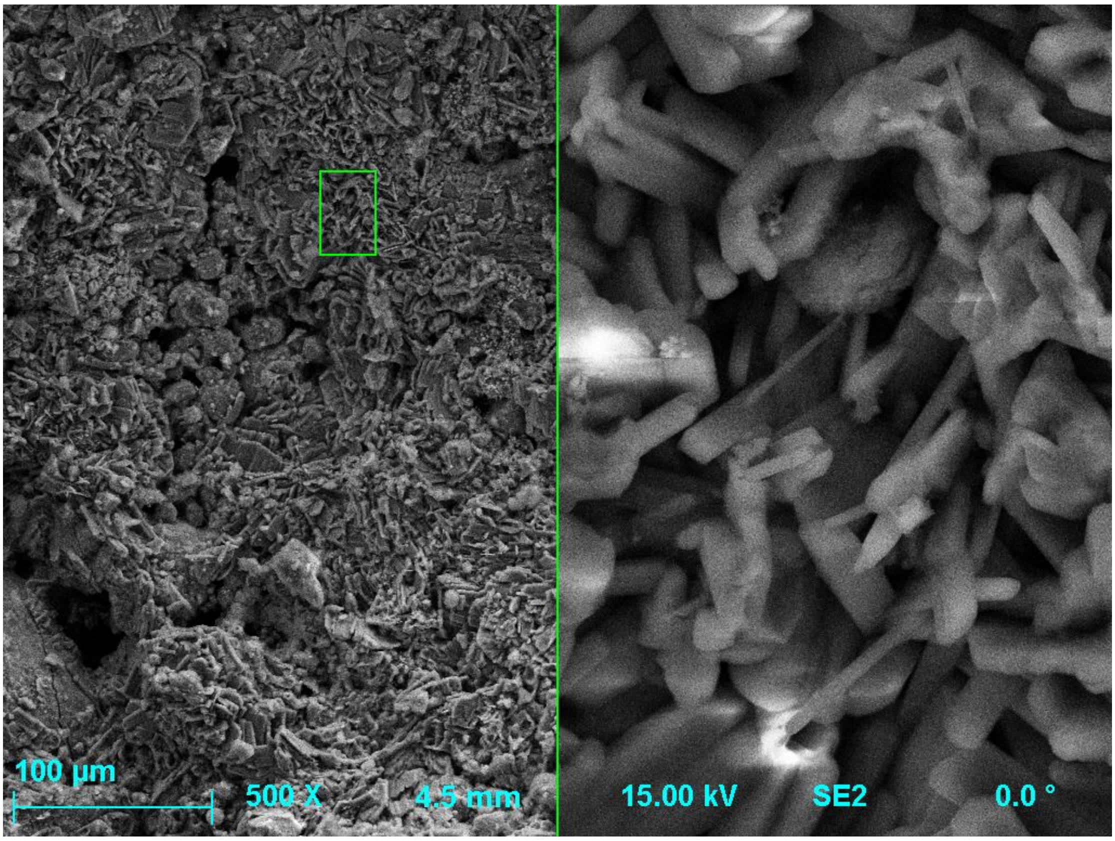

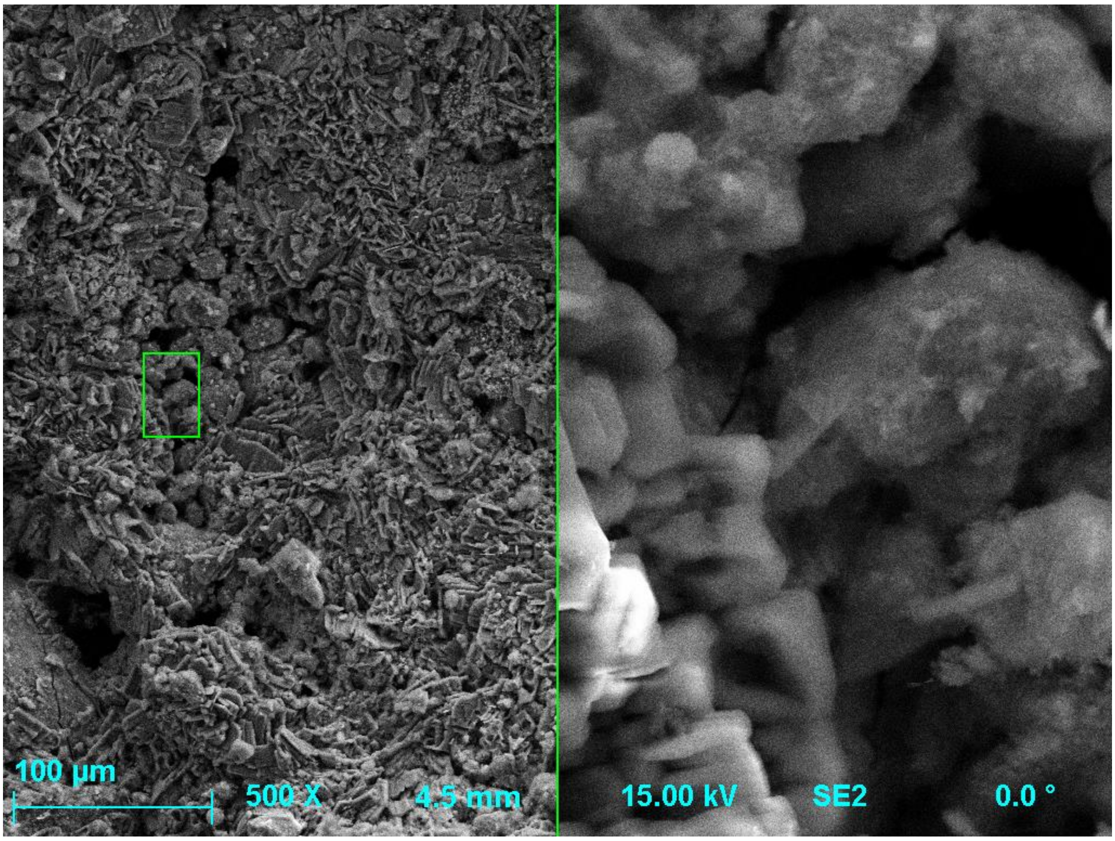

Images below show sizes of the smallest pores the instrument can resolve, around 40 nm.

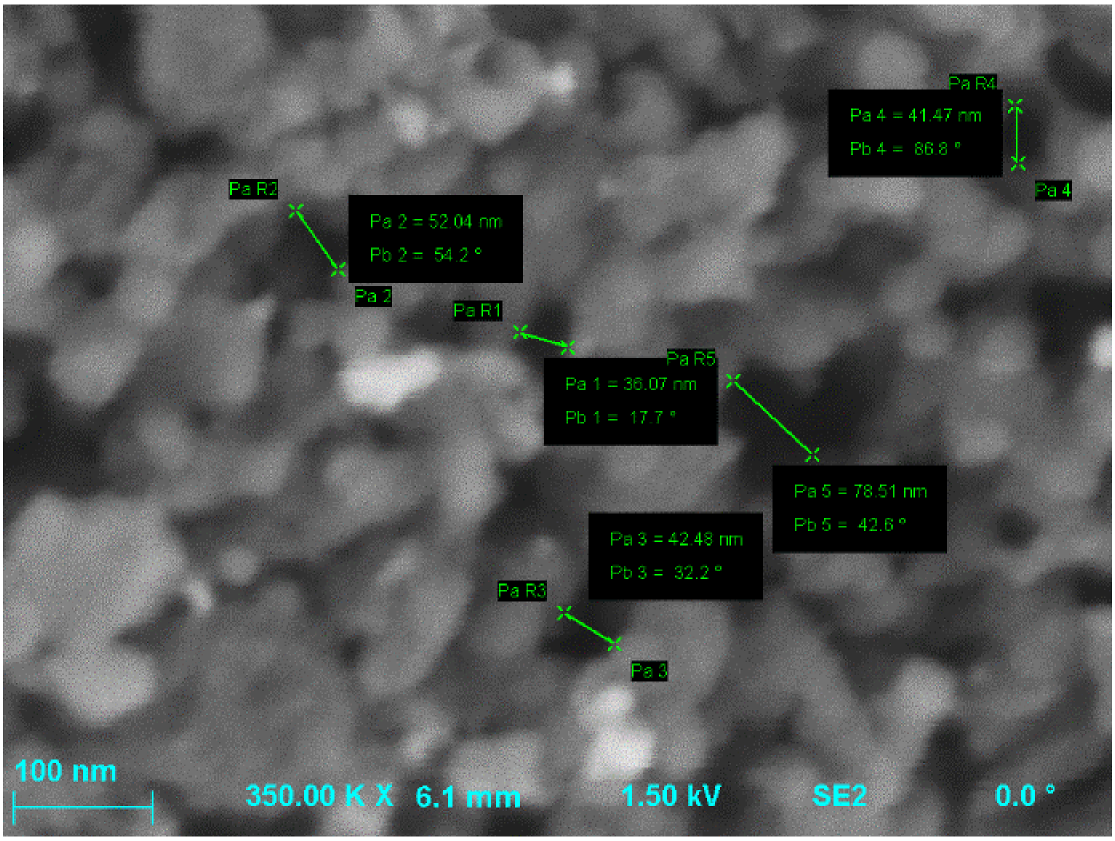

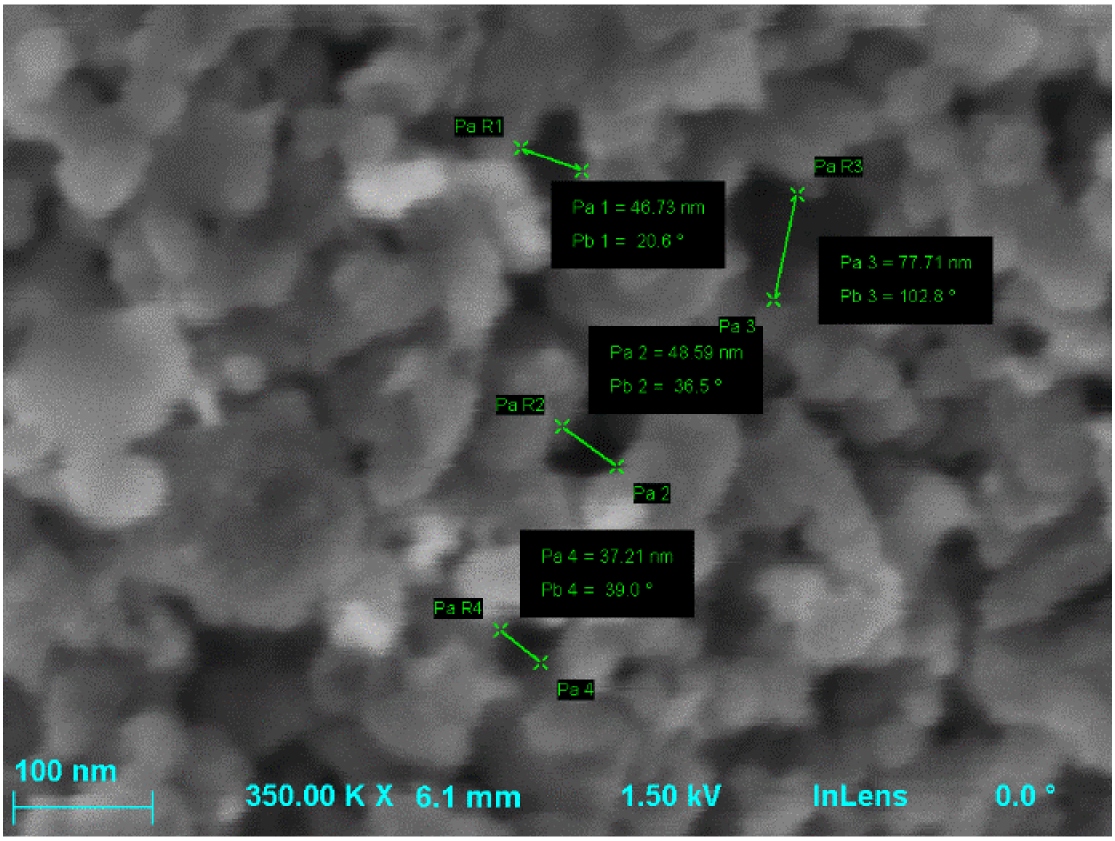

2. X-Ray Diffraction

The X-ray diffraction (XRD) analysis was performed using a diffractometer Bruker D2 PHASER. The analyzer is equipped with the following radiation source: Cu Kα at 30 kV and 10 mA. Material characterization was performed at 25°C temperature, with a step size of 0.015°, and an acquisition time of 1 s per step.

The XRD pattern of Fig. 1 indicates a broad, diffuse band in the 2θ range of $20° - 30°$, characteristic of amorphous carbon. A peak at $2\theta\sim 26°$ corresponds to the (002) plane of graphite, and another one at $2\theta \sim 43°$ is associated with the (100) plane of graphite. Peaks at 28°, 49°, and 56° likely arise from crystalline silicon nanoparticles (corresponding to the (111), (220), and (311) planes, respectively). Moreover, additional peaks at $23.1°$ and $26°$ may be indicative of sulfur-containing phases, which might be explained by impurities since the source carbon powder is waste-derived. Overall, while the dominant feature of the XRD pattern confirms a largely amorphous nature of the composite, the presence of crystalline contributions underscores a heterogeneous microstructure.

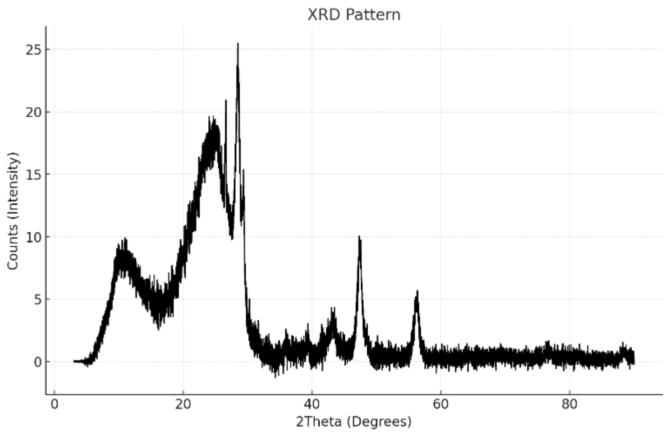

Figure 1: XRD pattern of the carbon-silica composite
